# Rac Regulates *Giardia lamblia* Encystation by Coordinating Cyst Wall Protein Trafficking and Secretion

**DOI:** 10.1128/mBio.01003-16

**Published:** 2016-08-23

**Authors:** Jana Krtková, Elizabeth B. Thomas, Germain C. M. Alas, Elisabeth M. Schraner, Habib R. Behjatnia, Adrian B. Hehl, Alexander R. Paredez

**Affiliations:** a Department of Biology, University of Washington, Seattle, Washington, USA; b Department of Experimental Plant Biology, Faculty of Science, Charles University in Prague, Prague, Czech Republic; c Institutes of Veterinary Anatomy and Virology, University of Zurich, Zürich, Switzerland; d Institute of Parasitology, University of Zurich, Zürich, Switzerland

## Abstract

Encystation of the common intestinal parasite *Giardia lamblia* involves the production, trafficking, and secretion of cyst wall material (CWM). However, the molecular mechanism responsible for the regulation of these sequential processes remains elusive. Here, we examined the role of GlRac, *Giardia*’s sole Rho family GTPase, in the regulation of endomembrane organization and cyst wall protein (CWP) trafficking. Localization studies indicated that GlRac is associated with the endoplasmic reticulum (ER) and the Golgi apparatus-like encystation-specific vesicles (ESVs). Constitutive GlRac signaling increased levels of the ER marker PDI2, induced ER swelling, reduced overall CWP1 production, and promoted the early maturation of ESVs. Quantitative analysis of cells expressing constitutively active hemagglutinin (HA)-tagged GlRac (HA-Rac^CA^) revealed fewer but larger ESVs than control cells. Consistent with the phenotype of premature maturation of ESVs in HA-Rac^CA^-expressing cells, constitutive GlRac signaling resulted in increased CWP1 secretion and, conversely, morpholino depletion of GlRac blocked CWP1 secretion. Wild-type cells unexpectedly secreted large quantities of CWP1 into the medium, and free CWP1 was used cooperatively during cyst formation. These results, in part, could account for the previously reported observation that *G. lamblia* encysts more efficiently at high cell densities. These studies of GlRac show that it regulates encystation at several levels, and our findings support its coordinating role as a regulator of CWP trafficking and secretion. The central role of GlRac in regulating membrane trafficking and the cytoskeleton, both of which are essential to *Giardia* parasitism, further suggests its potential as a novel target for drug development to treat giardiasis.

## IMPORTANCE

The encystation process is crucial for the transmission of giardiasis and the life cycle of many protists. Encystation for *Giardia lamblia* involves the assembly of a protective cyst wall via sequential production, trafficking, and secretion of cyst wall material. However, the regulatory pathways that coordinate cargo maturation and secretion remain unknown. Here, we asked whether the signaling activities of *G. lamblia*’s single Rho family GTPase, GlRac, might have a regulatory role in the encystation process. We show that GlRac localizes to endomembranes and its signaling activities regulate the production of cyst wall protein 1 (CWP1), the maturation of encystation-specific vesicles (ESVs), and secretion of CWP1. We also show that secreted CWP1 is available for the development of cysts at the population level, a finding that in part could explain why *Giardia* encystation proceeds more efficiently at high cell densities.

## INTRODUCTION

The diplomonad flagellate *Giardia lamblia* (syn., *G. intestinalis*, *G. duodenalis*) is the causative agent of giardiasis, a neglected human diarrheal disease (1). Annually, 280 million cases of this waterborne and foodborne disease are reported worldwide (2, 3). The *Giardia* life cycle consists of two stages: the parasitic trophozoite form, which colonizes the host’s upper intestine, and the water-resistant nonmotile infectious cyst form, which is shed in the host’s feces. Once *G. lamblia* leaves the host’s upper intestine, an increase in pH triggers encystation, leading to the stage differentiation of trophozoites to cysts (4, 5). This dormant form of the parasite features a protective wall which enables it to survive in the environment (6). Regulation of the encystation process is essential for the timely production of viable cysts and, ultimately, for the success of the parasite-host colonization strategy. In addition to *Giardia*, other protozoan parasites utilize an encystation strategy to maintain their life cycles (7). *Giardia* is currently the best-developed model for studying this process (8).

*Giardia* encystation involves pulsed production, processing, and secretion of large amounts of cyst wall material (CWM) (9, 10) which is composed of a fibrillar matrix containing three paralogous cyst wall proteins (CWP1 to 3) and a *Giardia*-specific β-1,3-GalNAc homopolymer (11–14). The encystation process takes roughly 24 h to complete and involves neogenesis of Golgi complex-like encystation-specific vesicles (ESVs), which have no equivalent in nonencysting trophozoites (15, 16). Trafficking of CWP begins with its accumulation in nascent ESVs at endoplasmic reticulum (ER) exit sites (ERES) (10), a process dependent on COPII and the small GTPase Sar1 (16). After approximately 8 h, most of the CWM is contained within ESVs. At this point, ESVs are no longer associated with COPII or Sar1 but are associated with Arf1, a GTPase whose activity as a molecular switch leads to the recruitment of the coat proteins COPI and clathrin (16). Arf1 activity is required for subsequent ESV maturation and CWP trafficking out of the cell (13, 16). ESVs are sites of CWP processing, which includes several posttranslational modifications (17–19). As ESVs mature they grow in size, and approximately 12 h into the encystation process, a major processing event leads to the formation of a condensed core with an outer fluid phase. Core condensation is catalyzed by cleavage of pro-CWP2 into N- and C-terminal fragments by a cysteine protease (20, 21). The fragments are then partitioned so that the fluid phase is composed of the N-terminal fragment of CWP2 in addition to CWP1, whereas the core contains CWP3 and the C-terminal part of CWP2. At the same time, a GalNAc carbohydrate homopolymer is produced and trafficked by distinct carbohydrate-positive vesicles that are deposited at the surface of encysting cells ahead of cyst wall protein secretion (22, 23). The curled fibrils of the GalNAc carbohydrate serve as a scaffold for direct binding of CWP1 via a lectin domain (22). The cyst wall is thought to be formed by a rapid secretion event that releases CWP1 and the N-terminal part of CWP2, likely within minutes, to form the first layer of the cyst wall; this is followed by slower deposition of the condensed core cargo, CWP3, and the C-terminal part of CWP2 (9). While considerable progress has been made in describing these sequential events of encystation, the signaling events which trigger secretion remain elusive. Since CWM secretion is necessary for the formation of environment-resistant infectious cysts, uncovering the mechanisms that regulate and temporally coordinate secretion of CWM could potentially identify potential drug targets of *Giardia* and also uncover conserved principles of protozoan encystation.

Rho GTPases are potential candidates for regulating CWP secretion, as they have important roles in coordinating vesicle trafficking and the cytoskeleton in plants and animals (24–28). Rho family GTPases have undergone extensive gene duplication and functional diversification in most eukaryotic lineages (copy number in humans, 22; in *Arabidopsis thaliana*, 11; in *Saccharomyces cerevisiae*, 5). However, the *Giardia* genome contains just a single Rho family GTPase, GlRac, and the entire signaling system appears to be minimalistic compared to that of mammals (see Table S1 in the supplemental material) (29–31). Interestingly, Rac has been reported to be the evolutionary founding member of the Rho family GTPases (32). Therefore, studies of *G. lamblia*, which is itself placed in a critical, albeit controversial, deep-branching position on the tree of life (33–35), may provide insight into the ancestral function of Rho family GTPases.

Here, we set out to determine if GlRac has a role in regulating membrane trafficking in *Giardia*. We report that GlRac is associated with the ER in trophozoites and with both the ER and the Golgi complex-like ESVs during encystation. GlRac is crucial for production of CWP1, maturation of ESVs, and most importantly, for secretion of CWP1, which is used in a cooperative manner to form viable cysts. These roles in *Giardia* indicate a conserved and ancient role for Rac homologs in membrane trafficking.

## RESULTS

### GlRac associates with the ER and encystation-specific vesicles.

**FIG 1:**
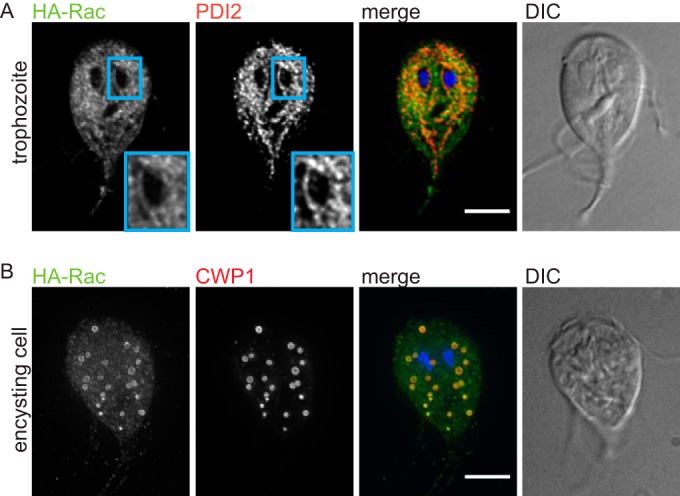
GlRac associates with the ER and ESVs. (A) Trophozoites were stained for HA-Rac (green), PDI2 (red), and DNA (blue) (merged image). HA-Rac staining partially overlapped with the ER marker PDI2. The inset shows enrichment of HA-Rac at the perinuclear ER. (B) Encysting cells were stained for HA-Rac (green), CWP1 (ESV marker; red), and DNA (blue). Bar = 5 µm. DIC, differential interference contrast images.

We previously observed that expression of a constitutively active GlRac mutant (tetracycline/doxycycline-inducible Q47L HA-Rac; HA-Rac^CA^, equivalent to Q61L Rac1 [see Fig. S1 in the supplemental material]), alters actin organization and, sometimes, results in formation of large vesicular structures in nonencysting trophozoites (31). The latter result suggested a possible role for GlRac in endomembrane organization. To further examine this possibility, we determined GlRac localization by endogenously tagging the protein at the N terminus with a triple hemagglutinin (HA) tag (HA-Rac). Localization of HA-Rac in trophozoites, the proliferative stage that colonizes the host intestine, revealed a pattern similar to that reported for the ER (36). We therefore examined its location relative to protein disulfide isomerase 2 (PDI2), an ER lumenal enzyme that catalyzes disulfide bond formation and protein folding (36). We observed considerable overlap between the two signals (Fig. 1A), indicating that a portion of GlRac is ER associated. To determine whether GlRac localization might be altered during the stage conversion to cysts, we induced encystation by exchanging standard medium for encystation medium and then examined the localization of HA-Rac 12 h into the encystation process. We found that HA-Rac was associated with the perimeter of CWP1-positive vesicles known as ESVs (Fig. 1B). The localization of GlRac to the ER and ESVs could indicate a role for Rac in regulating protein trafficking in *Giardia*.

### Constitutive GlRac signaling impairs ER function, CWP1 production, and cyst viability.

**FIG 2:**
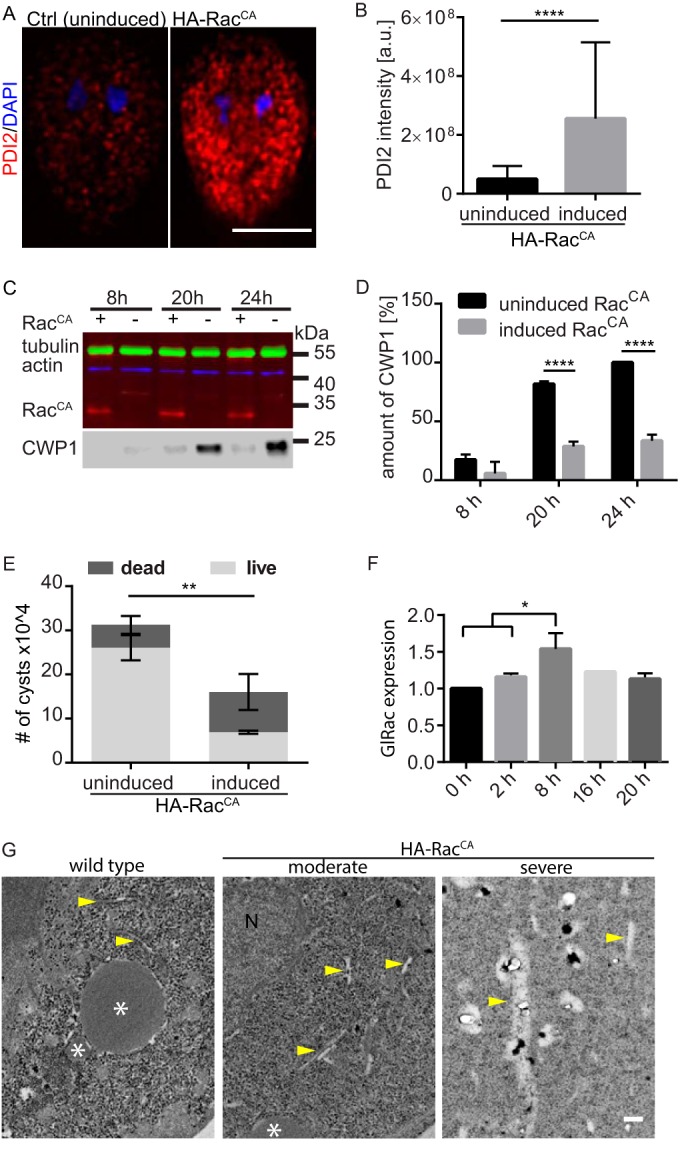
Constitutive GlRac signaling impairs ER function, CWP1 production, and cyst viability. (A) Expression of constitutively active GlRac resulted in increased PDI2 levels (red); representative images were acquired and scaled equally. Note that PDI2 levels in the control are similar to that of endogenously tagged Rac (see Fig. 1A); it was necessary to scale the image differently so that the induced HA-Rac^CA^ image would not be saturated. (B) Quantification of PDI2 levels. Statistical significance was evaluated from three independent experiments by *t* test (*n* = 45) for each condition: ****, *P* ≤ 0.0001. a.u., arbitrary units. Bar, 5 µm. (C) Western blot analysis showed decreased production of CWP1 in doxycycline-induced HA-Rac^CA^ encysting cells at 8, 20, and 24 h p.i.e. compared to production in the uninduced control. CWP1 was probed on the same blot after stripping. (D) Quantification of CWP1 levels from three independent experiments. Control cells at 24 h p.i.e. were set to 100%, and the relative amount of CWP1 was calculated based on normalization to the tubulin loading control. Statistical significance was evaluated by using the *t* test. ****, *P* < 0.0001. (E) Cyst production was quantified for induced HA-Rac^CA^ and control cells. Additionally, the cells were stained with the vital dye trypan blue to assay cyst wall integrity. Cysts production and viability data were acquired from three independent experiments, each with ≥200 cysts. Statistical significance was evaluated by using the *t* test. **, *P* < 0.01. (F) Relative expression of GlRac at 0, 2, 8, 16, and 20 h p.i.e. was tested by quantitative RT-PCR, for which *GAPDH* was used as a control. Three independent replicates, each consisting of three technical replicates, were evaluated by using the *t* test. *, *P* < 0.05. (G) TEM imaging of wild-type and doxycycline-induced HA-Rac^CA^ cells 13 h p.i.e. Yellow arrows, putative ER; N, nucleus; *, putative ESV. Bar, 200 nm.

To assess whether GlRac activity affects the ER, we tested the impact of inducing HA-Rac^CA^ expression on PDI2 localization 24 h after the addition of doxycycline (Fig. 2A). We observed a striking increase in PDI2 staining. Quantification verified a 4-fold increase in PDI2 levels as a result of induced HA-Rac^CA^ expression above that of the uninduced control (Fig. 2B). To verify a functional role for GlRac in encystation, we assessed the impact of constitutive GlRac signaling on CWP1 levels and the production and viability of cysts. We chose to follow CWP1 because it is the best-characterized cyst wall component and the only marker for which a commercial antibody is available. HA-Rac^CA^ expression led to over a 70% reduction in CWP1 levels 24 h into the encystation process, compared with its levels in uninduced control cells, as measured by quantitative immunoblotting (Fig. 2C and D). Consequently, the number of cysts formed by HA-Rac^CA^-expressing cells compared to the uninduced control was reduced by 49%. To compare the quality of the cyst walls formed by these two populations, we assessed the ability of the formed cysts to exclude the vital dye trypan blue after treatment with distilled water. Cysts with defective walls lyse in distilled water and stain blue. We observed a significant increase in trypan blue-positive cysts in the HA-Rac^CA^-expressing population (Fig. 2E).

Since misregulation of GlRac signaling impacted the ER, CWP1 levels, and cyst production, we questioned whether GlRac levels change during the encystation process. SAGE data in GiardiaDB reported that *GlRac* levels (GL50803_8496) decreased upon the initiation of encystation and then increased above the levels of nonencysting trophozoites 7 h into encystation (37). We used quantitative reverse transcription-PCR (RT-PCR) to analyze the time course of *GlRac* expression in wild-type (WT) cells at 0, 2, 8, 16, and 20 h post-induction of encystation (p.i.e.) relative to expression of the housekeeping gene *GAPDH* (38). In contrast to the SAGE and recently published transcriptome sequencing (RNA-Seq) data (8, 37), we did not observe reduced *GlRac* expression upon initiation of encystation, but we did observe that GlRac levels increased significantly at 8 h p.i.e. (Fig. 2F) (*P* < 0.05). Together, our studies indicate that GlRac is transcriptionally upregulated from ~7 to 12 h p.i.e.

While GlRac localizes to the ER, it may be mostly inactive there. Rho GTPases act as molecular switches by changing conformation based upon their nucleotide binding state. The active GTP-bound state functions by recruiting effectors to carry out specific activities, and it then becomes inactive after GTP hydrolysis and disassociation with effector proteins. Rho GTPases are activated by guanine nucleotide exchange factors (GEFs) through exchange of GDP for GTP (39). Therefore, the ER could be a source of sequestered GlRac positioned to associate with budding vesicles. In support of the notion that GlRac is largely inactive at the ER, constitutively active GlRac increased PDI2 levels, indicating ER stress (40). To further examine the impact of constitutive GlRac signaling on organization of the ER and other endomembranes, we analyzed the impact of HA-Rac^CA^ at the level of electron microscopy. In transmission electron micrographs (TEMs) of WT cells, ESVs are vesicles with a uniform electron density, and the ER appears as thin tubules (Fig. 2G; see also Fig. S2 in the supplemental material). In doxycycline-induced HA-Rac^CA^ encysting cells, we observed marked changes in endomembrane organization that likely correlated with the extent of HA-Rac^CA^ expression. In severely perturbed cells, we observed atypical ER morphology, with extensive swelling potentially indicating an ER exit defect. Our TEM analysis, in the absence of any markers, did not allow us to determine which if any of the vesicular structures observed in the most severely disrupted cells were ESVs. In any case, we observed electron-dense staining of vesicular content that we have not observed in wild-type controls (see Fig. S2 in the supplemental material). We interpret the electron-dense staining within vesicles to indicate defective CWM processing, and this may account for the reduction of CWP1 in HA-Rac^CA^-expressing cells. Taken together, we conclude that GlRac has a critical regulatory role in encystation, based on four observations: (i) GlRac associates with the ER and ESVs; (ii) GlRac is temporally upregulated during encystation; (iii) constitutive GlRac signaling causes ER swelling and reduces cellular CWP1 levels; and (iv) constitutive GlRac signaling reduces encystation rates as well as disrupts cyst wall integrity.

### GlRac promotes ESV maturation.

**FIG 3:**
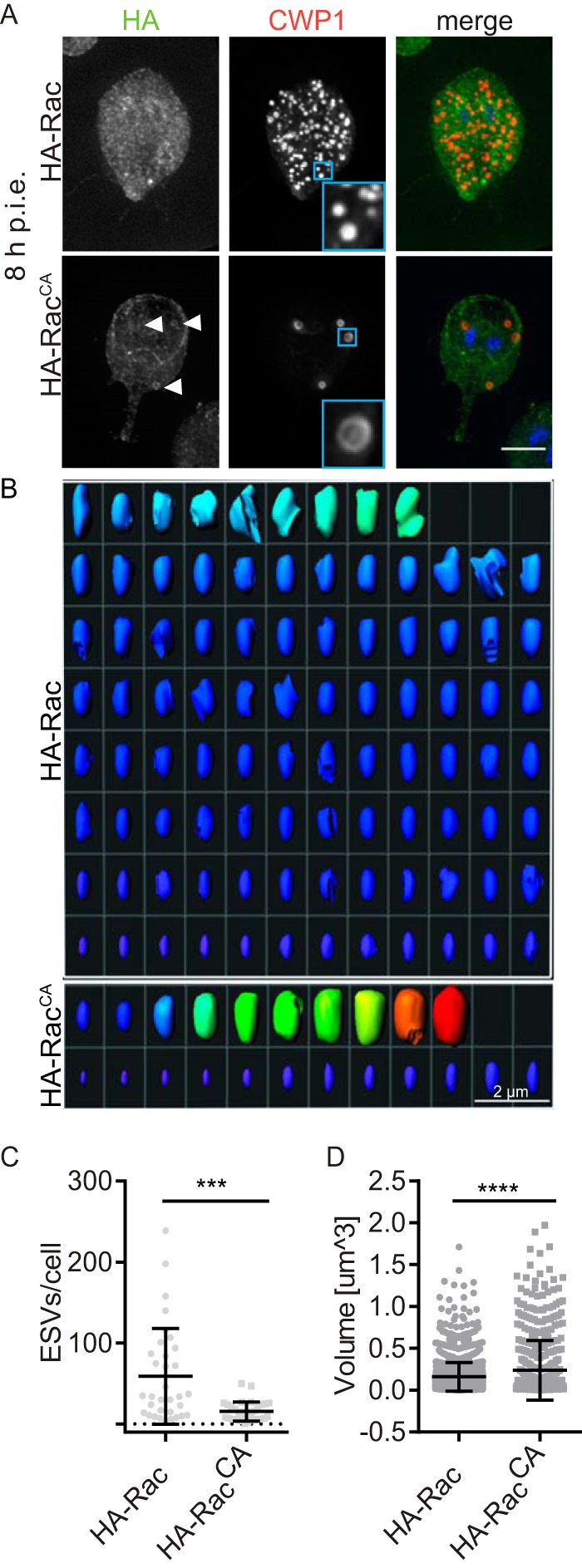
GlRac promotes ESV maturation. (A) Endogenously tagged HA-Rac and doxycycline-induced HA-Rac^CA^ cells were stained for HA (green) and CWP1 (red) at 8 h p.i.e. Maturation of ESVs is indicated by the ring-like distribution of CWP1 (inset) in doxycycline-induced HA-Rac^CA^ cells but not in cells expressing endogenously tagged HA-Rac. The ring-like distribution results from core condensation, which pushes CWP1 to the perimeter of the ESV. Arrowheads indicate HA-Rac^CA^ localized on ESVs. Bar, 5 µm. See also Fig. S3 in the supplemental material. (B) ESVs from a representative endogenously tagged HA-Rac cell (the cell has the average number of ESVs for the WT HA-Rac population) and a doxycycline-induced HA-Rac^CA^ cell (the cell has an average number of ESVs for the HA-Rac^CA^ population) viewed after 3D segmentation analysis. The heat map highlights ESV size: warmer colors are larger. Bar, 2 µm. (C) Quantification of ESVs. (D) Quantification of ESV volumes. At least 33 cells under each condition were analyzed from three independent replicates. Statistical significance was evaluated by using the *t* test. ****, *P* < 0.0001; ***, *P* < 0.001.

To better understand the temporal role of GlRac in encystation, we encysted doxycycline-induced HA-Rac^CA^ and the endogenously tagged HA-Rac cell lines. Cells were collected every 2 h from 2 to 24 h p.i.e. and stained for CWP1 and HA (see Fig. S3 in the supplemental material). Consistent with the results described above, in which GlRac expression levels were shown to increase by 8 h p.i.e., we observed a striking difference between HA-Rac and HA-Rac^CA^ cells at 8 h p.i.e. ESVs in HA-Rac^CA^-expressing cells were fewer, larger in volume, and more often displayed a ring-like CWP1 distribution than did the HA-Rac control (Fig. 3A; see also Fig. S3). The ring-like CWP1 distribution is characteristic of mature ESVs that have undergone processing and condensation and is not typically observed until 12 to 16 h p.i.e. (9). We quantified the number and volume of ESVs in the cells via three-dimensional (3D) image segmentation. The number of ESVs formed per cell was reduced by 73.6% in HA-Rac^CA^-expressing cells compared to HA-Rac cells (Fig. 3B and C). Additionally, the average volume of individual ESVs was 50% larger in HA-Rac^CA^-expressing cells than in HA-Rac cells (Fig. 3B and D). Due to leaky expression (see Fig. S4A in the supplemental material), uninduced HA-Rac^CA^ cells displayed an intermediate phenotype (see Fig. S4B and C), which is why we used HA-Rac cells as a negative control here. Nevertheless, the doxycycline-induced HA-Rac^CA^ cells had a significant increase in volume over uninduced HA-Rac^CA^ cells. These findings indicate that GlRac signaling can promote increased ESV size and precocious maturation, a result that corresponds well with its transcriptional increase at 7 to 12 h p.i.e. in wild-type *Giardia*.

### Rac drives secretion of CWP1, which can be utilized by the entire population of cysts.

**FIG 4:**
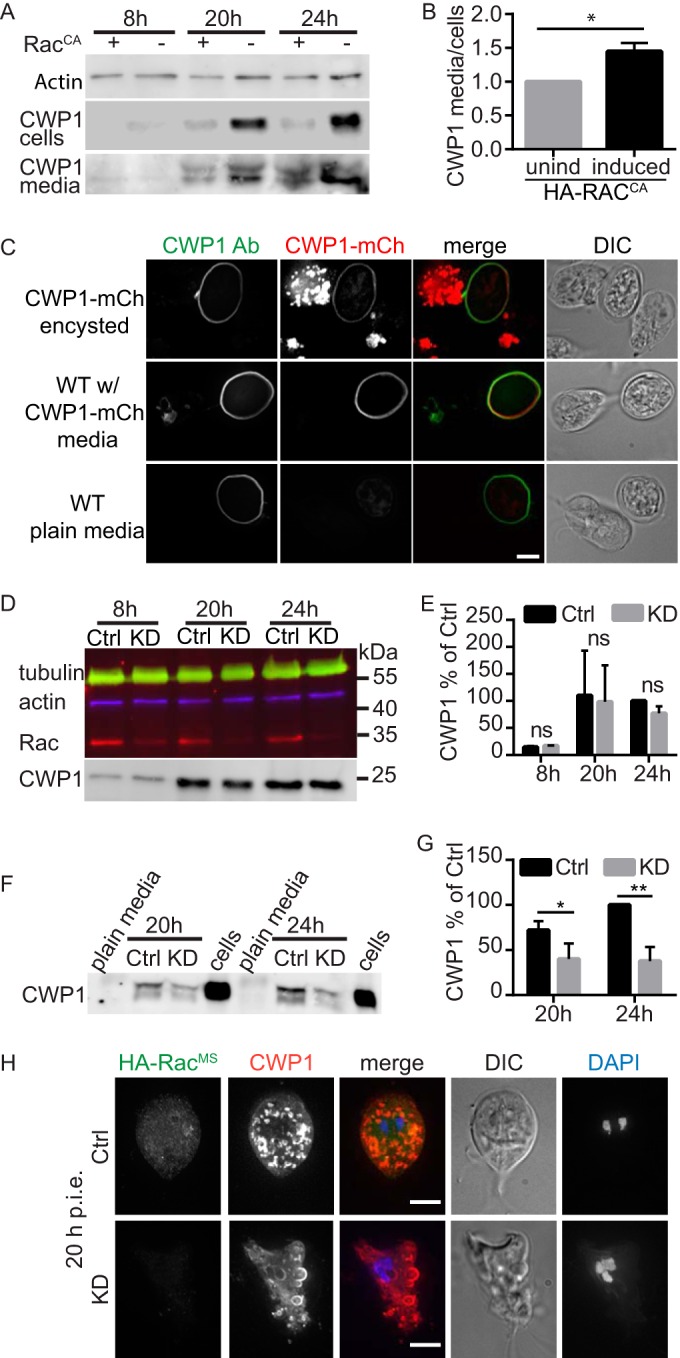
GlRac drives secretion of CWP1 that can be utilized by other cysts in the population. (A) Western blot showing CWP1 levels in cells and the media they were grown in for both doxycycline-induced HA-Rac^CA^ and uninduced control cells at 8, 20, and 24 h p.i.e. (B) The ratio of CWP1 secreted into the medium versus that found in cells was quantified at 20 h p.i.e. from three independent experiments (levels for cells and media were first normalized to the uninduced control before calculating the ratio). (C) Free CWP1 detected in the medium can be used by the entire population. Sterile filtered medium from encysting CWP1-mCherry-expressing cells at 24 was replaced with the medium of WT cells. After a subsequent 24 h in the CWP1-mCherry medium, 100% (50/50) of the WT cysts were CWP1-mCherry positive, while 0/50 WT cells encysted after 48 h displayed red fluorescence. (D) Quantitative Western blot showing CWP1 levels after depleting GlRac with translation-blocking morpholinos (see Fig. S6B in the supplemental material). (E) Quantification of results from three independent experiments, indicating that GlRac depletion does not significantly impair the production of CWP1 in encysting cells after 8, 20, and 24 h p.i.e. (CWP1 in control cells at 24 h p.i.e. was normalized to 100%; ns, nonsignificant). (F) Analysis of CWP1 levels in the medium of morpholino-treated cells. Note that plain encystation medium was loaded as a negative control and lysates of encysting cells were loaded as positive controls. (G) Quantification of secreted CWP1 from three independent experiments (CWP1 levels in control cells at 24 h were normalized to 100%). (H) Immunofluorescence localization of HA-Rac^MS^ (green) and CWP1 (red) were analyzed at 20 h p.i.e. Note that ESVs in KD cells appear to be larger and stuck beneath the plasma membrane (see also Fig. S6A in the supplemental material). Bar, 5 µm. Statistical significance was evaluated with the *t* test. **, *P* ≤ 0.01; *, *P* ≤ 0.05.

Cyst wall formation is thought to be temporally regulated, because CWM is held in the Golgi complex-like ESVs for processing and sorting before its concerted secretion to form the cyst wall (9). Our observations of precocious ESV maturation and reduced intracellular CWP1 may be consistent with a switch from regulated to constitutive secretion of CWM. Therefore, we expected that if HA-Rac^CA^ caused secretion in a temporally uncontrolled manner, we would detect free CWP1 in the medium, as secreting cells would not yet be competent to bind CWP1. This was indeed found to be the case. Cells expressing HA-Rac^CA^ displayed an increased ratio of CWP1 exported into the medium compared to the CWP1 remaining in the cells (Fig. 4A and B). While we had expected to find free CWP1 in the medium of HA-Rac^CA^-expressing cells, we were surprised to find such high levels of CWP1 in the medium of the control. This prompted us to ask whether the CWP1 detected in the medium was functional (i.e., could be incorporated into the cyst wall of any cell in the population), a possibility that would implicate encystation as a cooperative process. Furthermore, cooperative production of CWP1 might in part account for previous observations that encystation rates improve with greater parasite densities (5). To test this, we conducted a medium swap experiment. Beginning with confluent cultures (~1 × 10^6^ cells/ml), CWP1-mCherry-expressing cells or WT cells were incubated in encystation medium for 24 h. The medium in which CWP1-mCherry cells were encysted (CWP1-mCherry medium) was cleared of cells by centrifugation and filtration (0.22 µm) before being used to replace the medium of encysting WT cells. Following incubation for a further 24 h, we found that 100% (50/50) of the WT cysts were mCherry positive and, as expected, 0/50 WT cells from the plain medium condition had detectable red fluorescence under identical image acquisition and scaling (Fig. 4C). Consistent with the idea that only cells at a specific stage (i.e., the cells at later stages of encystation, displaying GalNAc homopolymers) are competent to bind CWP1 (22), we only found mCherry-CWP1 on rounded-up late-stage encysting cells or cysts and never on trophozoite-shaped cells (Fig. 4C). These results indicate that secreted CWP1 can be utilized at the population level and help to start explaining the improved encystation rates associated with greater parasite density (5).

To further validate the role of GlRac in secretion, we sought to inhibit GlRac signaling through loss-of-function. The ability to knock out genes in *Giardia* with genome editing has yet to be accomplished, and RNA interference is ineffective. Therefore, we sought to deplete GlRac by using translation-blocking morpholinos (41). In order to monitor GlRac protein levels without a custom antibody, we developed an endogenously tagged morpholino-sensitive HA-GlRac construct (HA-Rac^MS^) by including the first 27 bp of the Rac coding region in front of the HA tag (see Fig. S5 in the supplemental material). Without this short sequence, the 5′ HA tag would render the tagged copy of GlRac morpholino resistant (42). HA-Rac^MS^ localization was indistinguishable from that of the previously used endogenously tagged HA-Rac (see Fig. S5D). Western blot analysis confirmed that GlRac expression was reduced by 72% at 24 h after morpholino electroporation (see Fig. S5). Depletion of GlRac in trophozoites led to defects in morphology, polarity, and cytokinesis (see Fig. S5D). In agreement with the notion that GlRac activity is not required for the initial stages of encystation, GlRac depletion did not cause an appreciable change in cellular CWP1 content (Fig. 4D and E); it did, however, impair CWP1 secretion, as indicated by the significantly decreased amount of CWP1 detected in the medium during the later stages of encystation (Fig. 4F and G; see also Fig. S6 in the supplemental material). Consistent with this phenotype, we frequently observed cells with ESVs persisting at the periphery of the cells. We interpret this finding to indicate that ESV fusion with the plasma membrane was impaired and thus the CWM cargo remained trapped in the cell (Fig. 4H; see also Fig. S6).

Taken together, our findings demonstrate two major functions for GlRac in encystation. GlRac signaling promotes an increase in ESV size and maturation and is required for CWP1 secretion.

## DISCUSSION

We set out to determine whether GlRac plays a critical role in regulating membrane trafficking and, more specifically, if it has a role in regulating the trafficking of CWM. The initial production of CWM is dependent on the ER (10). Enrichment of GlRac at the ER in nonencysting and encysting trophozoites (Fig. 1) is consistent with a role in regulating ER structure and/or function. CWP1 release from the ER depends on functional ERES and is a necessary first step in ESV biogenesis (10). The accumulation of PDI2 and the swollen ER phenotype associated with HA-Rac^CA^ expression suggest an ER exit defect (Fig. 2B and G; see also Fig. S2 in the supplemental material). Whether GlRac has a regulatory role for ERES remains to be elucidated. Interestingly, however, upregulation of GlRac expression coincides with the completion of CWP loading into nascent ESVs around 8 h p.i.e. (16). There is also precedence for Rho GTPase regulation of ERES. SPIKE1, an *Arabidopsis* guanine nucleotide exchange factor (GEF), localizes to ERES, where it activates the Rac homolog Rho of plants 2 (AtROP2) (29, 43). Expression of AtROP2 dominant-negative or constitutively active mutants resulted in upregulation of ER stress response genes (43), which is similar to our observation that constitutive GlRac signaling increased PDI2 levels, a known ER stress response (40). We speculate that constitutive GlRac signaling caused ER trafficking defects that resulted in reduced CWP1 levels as a consequence of overall reduced capacity to produce and sort CWP1.

Precedent also exists for Rho family GTPases controlling secretory traffic through coordination of the cytoskeleton and membrane trafficking systems (25). We previously demonstrated that GlRac has a role in regulating the actin cytoskeleton in *Giardia* (31). Furthermore, actin was shown to localize to late-stage ESVs (31). Since GlRac is enriched around ESVs (Fig. 1B), it is tempting to speculate that GlRac works to coordinate the cytoskeleton and membrane trafficking systems during ESV maturation and secretion. For example, GlRac could trigger actin-based translocation of smaller ESVs in order to bring them into proximity for fusion. A general role in coordinating the cytoskeleton and membrane trafficking systems would be in line with Rho GTPase functions in other eukaryotes. Considering that Rac is the evolutionary founding member of the Rho family GTPases (32) and that *Giardia* belongs to a potentially early-branching group of eukaryotes (34, 44), our results suggest that the ancestral Rac homolog was capable of regulating multiple processes and that the functional diversification of the expanded Rho family GTPases is a modern adaptation.

### Model of GlRac regulation of encystation.

**FIG 5:**
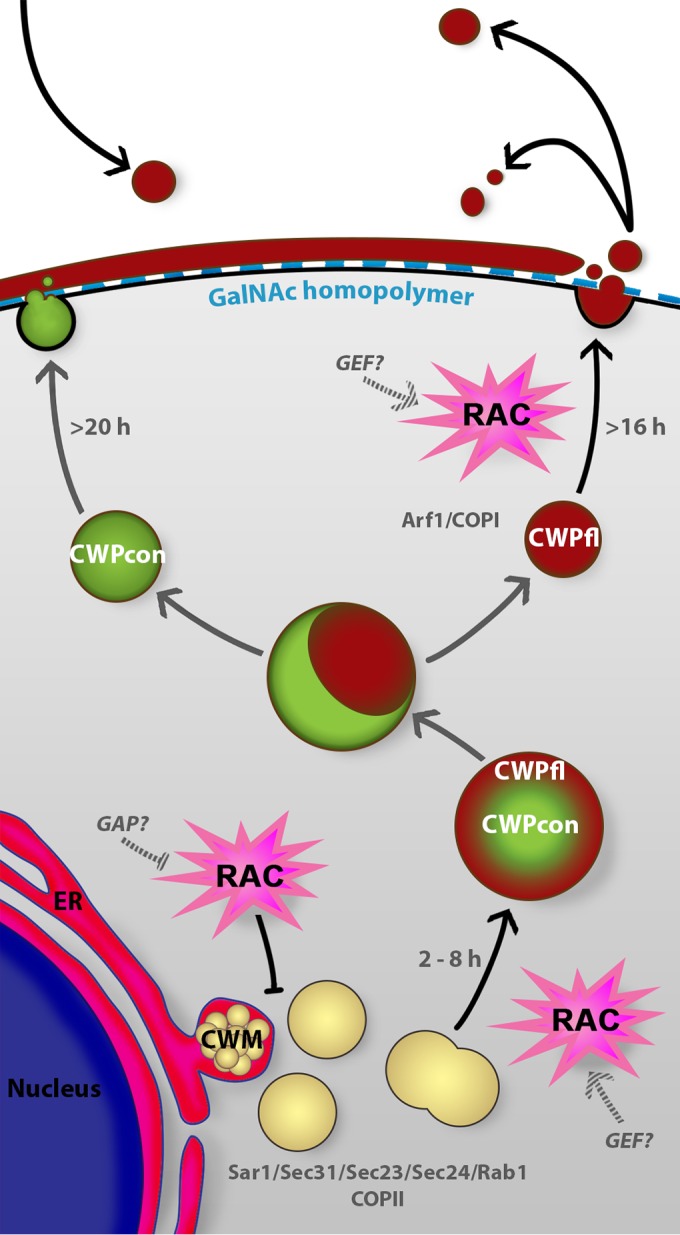
Model of GlRac regulation of encystation. Based on our findings from this study, we propose a model of encystation where GlRac coordinates the encystation process. Newly synthesized CWM is produced in the ER and exported from the ER into nascent ESVs (15, 46). GlRac signaling disrupts export from the ER, a process dependent on active ERES and the small GTPase Sar1 (10, 16). Active GlRac drives maturation of ESVs (black arrows). Mature ESVs are composed of the condensed core (con), containing CWP3 and the C-terminal part of processed CWP2, and the fluid outer phase (fl), composed of CWP1 and the N-terminal part of processed CWP2 (9). Maturation of ESVs is a consequence of CWP2 processing by a cysteine protease (20). Double-phase ESVs decondense to be sorted and sequentially secreted. Ahead of CWP secretion, GalNAc homopolymer is exported to the cyst surface by distinct carbohydrate-positive vesicles (ECVs) (22, 23). CWP1 secretion is regulated by GlRac signaling (black arrows) and is crucial to form the first protein layer of the impermeable cyst wall. CWP1 binds the GalNAc carbohydrate fibrils present at the surface of the encysting cell (22) but can also diffuse away and be bound by other “primed” encysting cells (black arrows). By affecting CWP1 levels in the cell, temporal regulation of ESVs maturation, and CWP1 secretion, GlRac signaling coordinates the sequential CWP1 production and trafficking events necessary for the production of viable cysts.

*Giardia*’s parasitic life cycle is dependent on stage conversion from trophozoites to infectious water-resistant cysts, which preserves their viability in the outer environment and passage through stomach acid. Encystation features sequential vesicle trafficking events and pulsed secretion of CWM to the surface of the newly formed cyst (9). Our results and the current understanding of the process are summarized in Fig. 5, an updated model for encystation (9, 45). Newly produced CWPs destined to form the impermeable cyst wall are produced in the ER and sorted into nascent ESVs (15, 46). ESV neogenesis is dependent on functional ERES (10), which use the small GTPase Sar1 and COPII to form export vesicles (13, 16). Our data suggest that Rac signaling is inhibitory at this stage of trafficking (Fig. 2; see also Fig. S3 in the supplemental material). During early encystation (around 8 to 12 h p.i.e.), we observe the transition from many small ESVs to fewer larger ESVs; this process is promoted by GlRac signaling (Fig. 3). Coincidentally, Arf1 begins to associate with ESVs, and its activity is required for ESV maturation and ultimately CWP secretion (13, 16), around the same time GlRac transcript levels rise. It is tempting to speculate that these events are related, as cross talk between Arf and Rho GTPases has been observed for Arf and Rho GTPase homologs in plants and animals (reviewed in references 47 and 48). CWM processing occurs during the transition to larger ESVs and is apparent as an outer fluid phase and an inner condensed phase. The outer fluid phase is composed of CWP1 and the N-terminal processed portion of CWP2, while the condensed core is composed of CWP3 and C-terminal part of CWP2 (9). The mechanism by which GlRac signaling promotes an increase in ESV volume and concurrent CWM processing remains to be elucidated. We speculate that the mechanism will involve fusion of early ESVs and vesicles containing the protease responsible for the proteolytic cleavage of CWP2 (20, 21). During the later stages of encystation, in a second sorting event, the fluid and condensed phases of mature ESVs are separated and secreted in sequential steps to form a double-layered cyst wall (9). First, the fluid phase, composed of CWP1 and the N-terminal region of CWP2, is secreted in a GlRac-dependent manner (≥16 h p.i.e.). CWP1 has been proposed to contain a lectin domain that interacts with GalNAc homopolymer fibrils present at the surface of the forming cyst (22). Our results indicate that this is a cooperative process where CWP1 cannot only bind the cell it is secreted from but also contribute toward cyst wall formation of neighboring encysting cells (Fig. 5). In the final stages of encystation (≥20 h p.i.e.), the cyst wall is “sealed” with CWP3 and the C-terminal part of CWP2; these proteins are exported from the subpopulation of ESVs that inherited condensed cores (9). Overall, *Giardia*’s sole Rho family GTPase appears to play a central role in coordinating maturation and secretion of CWP1. We hypothesize that GlRac’s roles in ESV maturation (Fig. 3), formation of aberrant vesicles (Fig. 4) (31), and CWP secretion (Fig. 5) could all be explained by a role for GlRac in promoting membrane fusion. The specific effectors activated by GlRac signaling remain to be identified.

### Conclusion.

Our data indicate that the sole Rho family GTPase in *Giardia* has a critical role in regulating the encystation process. GlRac is key to temporal coordination of CWP1 production, ESV maturation, and CWP1 secretion, all of which are required for cyst formation and viability. The relative simplicity of *Giardia*’s membrane trafficking and Rho GTPase signaling system, whether evolutionarily ancient or derived, may be a powerful tool for uncovering core principles of cellular signaling, membrane trafficking, and the encystation process. In addition, Rho GTPases are druggable targets (49, 50), so the modulation of GlRac activity may be a potent tool to suppress *Giardia* infection and transmission. Indeed, rational drug design has been successfully used to target human Rac1 while sparing the related Rho family GTPases RhoA and Cdc42 (49–51). Since human Rac1 is more similar to human Cdc42 (70% identity) than to GlRac (50% identity), this divergence should allow for *Giardia*-specific inhibitors to be developed, and thus we suggest GlRac as a promising new candidate for drug development.

## MATERIALS AND METHODS

### Strain and culture conditions.

*G. lamblia* strain WB clone C6 (ATCC 50803) was cultured as described in reference 52. Knockdown experiments were performed as described elsewhere (41) using the Rac morpholino oligonucleotide 5′ TATCCTCATTTCCTGTACTAGTCAT 3′ and a control (Ctrl) morpholino oligonucleotide (5′ CCTCTTACCTCAGTTACAATTTATA 3′). For transfection, 5 to 50 µg DNA was electroporated (375 V, 1,000 µF, 750 Ω; GenePulser Xcell; BioRad, Hercules, CA) into trophozoites. Expression of Rac^CA^ was induced with 10 or 20 µg/ml doxycycline hydrochloride 12 to 24 h before the start of experiments.

### Encystation and viability assay.

To allow observation of different encystation stages, cells were synchronized by using the two-step encystation protocol (53). Confluent cultures of ~1 × 10^6^ cells/ml were cultivated for 24 h in preencystation medium (without bile). Encystation was induced as described previously (54) in medium with a pH of 7.8 and supplemented with 10 g/liter bovine/ovine bile. Trophozoites were encysted 12 to 24 h post-morpholino treatment or induction of Rac^CA^ expression. Two days after water treatment, cysts were stained with trypan blue and counted by using a hemocytometer.

### Vector construction.

Construction of the tetracycline-inducible WT and *Q74L HA-GlRac* (HA-Rac^CA^) vectors is described in reference 31. N-terminally HA-tagged *GlRac* (GL50803_8496) and morpholino-sensitive *GlRac* under endogenous promoters (HA-Rac^MS^) were constructed as follows. The HA*-Rac* fragment was cleaved with NcoI and EcoRI from a tetHA-Rac.pac vector and ligated into pKS-HA.pac enriched with an NcoI site. The NcoI restriction site was introduced into the pKS-HA.pac vector by using adapter oligonucleotides NcoI F′ (GATCCCCATGGG) and NcoI R′ (AATTCCCATGGG). To create an N-terminally tagged endogenously expressed HA*-Rac* that would remain sensitive to morpholinos, we introduced the first 27 bp of the Rac coding sequence before the HA tag (see Fig. S5A in the supplemental material). Native promoter-only or native promoter plus the first 27 bp (morpholino binding site for Rac^MS^) of the *Rac* gene flanking XbaI and NcoI sites was PCR amplified from *Giardia* genomic DNA using the primers XbaRacprom_F (5′ AAATCTAGAGTGCCGAGGCGGGATTGCTC 3′) and RacpromNcoI_R (5′ AAACCATGGTTTTTAATTTTTGTAACATG 3′) for GlRac and XbaRacprom_F (5′ AAATCTAGAGTGCCGAGGCGGGATTGCTC 3′) and NcoRacMORF_R′ (5′ ATTCCATGGCTGTATCCTCATTTCCTGTAC 3′) for Rac^MS^, respectively. The HA-Rac^MS^ plasmid was linearized for homologous recombination into the *Giardia* genome by using PstI.

To construct *CWP1*-mCherry, pKS-3HA.neo was digested with BamHI and EcoRI to remove the 3× HA tag. *CWP1*, including endogenous promoter sequence, was PCR amplified from *Giardia* genomic DNA by using the primer CWP1prom_F (5′ AAAGGATCCAAGCTTCTAGCCACGCATGGGCTG 3′) with flanking BamHI site and the CWP1_R (5′ CCGACCGGTccCCAAGGCGGGGTGAGGCAG 3′) primer with flanking AgeI site. mCherry was PCR amplified from a donor vector by using mCherry_F (5′ GGGACCGGTTGGAGGCGGAGGGAGCGGCGGGGGCGGAAGCATGGTGAGCAAGGGCGAG 3′) primer flanking AgeI site and a spacer to ensure proper folding of the fusion protein and mCherry_R (5′ AAAGAATTCTCACTTGTACAGCTCGTCC 3′) primer flanking the EcoRI site. The three fragments were ligated, and the sequence was verified before being transfected for episomal expression.

### RT-PCR.

RNA from encysting WT cells at the indicated times post-induction of encystation was isolated using the Illustra RNAspin kit (GE Healthcare Life Sciences, Pittsburgh, PA). RNA was reverse transcribed to cDNA by using the iScript cDNA synthesis kit (Bio-Rad, Hercules, CA). Quantitative PCR (qPCR) was performed using SsoAdvanced Universal SYBR Green supermix (Bio-Rad, Hercules, CA), and primers were designed using Primer3 software as follows. For Rac cDNA, primers qPCR RAC F2 (5′ GTGCAGAGGAAGTTGCAAAAG 3′) and qPCR RAC R12 (5′ GCGGATTGCACTATCAAACA 3′) were used. For glyceraldehyde 3-phosphate dehydrogenase (GAPDH) housekeeping gene cDNA (GL50803_6687), used as a control (38, 55), primers qPCR GAP1 F2 (5′ CAAGGGGATCATGACCTACAC 3′) and qPCR GAP1 R2 (5′ AGGCAACCAGCTTAACGAAC 3′) were used.

### Transmission electron microscopy.

For TEM, cells attached to carbon-coated sapphire disks were fixed with 2.5% glutaraldehyde in 0.1 M Na/K-phosphate (pH 7.4) for 1 h, washed with 0.1 M Na/K-phosphate, postfixed with 1% osmium tetroxide in 0.1 M Na/K-phosphate for 1 h, and dehydrated in a series of ethanol starting at 70%. After two changes in acetone, the processed cells were embedded in Epon at room temperature followed by polymerization at 60°C for 2.5 days. Sections of 60 to 80 nm in thickness were stained with uranyl acetate and lead citrate and analyzed in a transmission electron microscope (CM12; FEI, Eindhoven, the Netherlands) equipped with a charge-coupled-device camera (Ultrascan 1000; Gatan, Pleasanton, CA) at an acceleration voltage of 100 kV.

### Immunofluorescence microscopy.

For the immunofluorescence microscopy, cells were pelleted at 500 × *g* at room temperature and the pellet was fixed in PME [100 mM PIPES (pH 7.0), 5 mM EGTA, 10 mM MgSO_4_; PIPES is an abbreviation for piperazine-*N*,*N*′-bis(ethanesulfonic acid)] supplemented with 2% paraformaldehyde (Electron Microscopy Sciences, Hatfield, PA), 100 µM 3-maleimidobenzoic acid *N*-hydroxysuccinimide ester (Sigma-Aldrich), 100 µM ethylene glycol bis(succinimidyl succinate) (Pierce), and 0.025% Triton X-100 for 30 min at 37°C. Cells were washed two times in PME and then adhered to coverslips coated with poly-l-lysine (Sigma-Aldrich). The cells were washed again and permeabilized with 0.1% Triton X-100 in PME for 10 min. After two washes with PME, the cells were blocked for 30 min in PMEBALG (PME plus 1% bovine serum albumin, 0.1% NaN_3_, 100 mM lysine, 0.5% cold water fish skin gelatin [Sigma-Aldrich]). Rabbit anti-*Giardia* actin (GlActin) antibody 28PB+1 (31) and anti-HA mouse monoclonal HA7 antibody (Sigma-Aldrich) were both diluted 1:125 in PMEBALG, and cells were incubated in antibody solution overnight. After three washes with PME plus 0.05% Triton X-100, Alexa 488-conjugated goat anti-mouse and Alexa 555-conjugated goat anti-rabbit (Molecular Probes) secondary antibodies were diluted 1:200 in PMEBALG, and cells were incubated in these mixtures for 2 h. After three washes with PME plus 0.05% Triton X-100, the cells were postfixed in PME plus 1% paraformaldehyde and 0.025% Triton X-100 for 15 min, briefly washed three times in PME plus 0.05% Triton X-100, blocked in PMEBALG for 30 min, and incubated in 1:200 Alexa 647-conjugated anti-CWP1 antibody (Waterborne, New Orleans, LA) for 2 h. After three washes with PME plus 0.05% Triton X-100, coverslips were mounted with ProLong Gold antifade plus 4′,6-diamidino-2-phenylindole (DAPI; Molecular Probes). Anti-PDI2 antibody was used to detect the ER (36, 56). A Zenon mouse IgG labeling kit (Life Technologies, CA, USA) was used to costain HA-Rac and PDI2. Fluorescent images were acquired on a DeltaVision Elite microscope using a 100×, 1.4-numerical aperture objective and a PCO Edge sCMOS camera. Deconvolution was performed with SoftWorx (API, Issaquah, WA).

### Protein analysis and immunodetection.

*Giardia* parasites were harvested for protein analysis and immunodetection after chilling the culture tubes in ice for 30 min. After detachment, cells were pelleted at 700 × *g* and washed once in HEPES-buffered saline. To detect secreted CWP1, the medium was filtered, denatured in sample buffer, and boiled. The cells were resuspended in 300 µl of lysis buffer (50 mM Tris [pH 7.5], 150 mM NaCl, 7.5% glycerol, 0.25 mM CaCl_2_, 0.25 mM ATP, 0.5 mM dithiothreitol, 0.5 mM phenylmethylsulfonyl fluoride, 0.1% Triton X-100, Halt protease inhibitors [Pierce]) and sonicated. The lysate was cleared by centrifugation at 10,000  × *g* for 10 min at 4°C and then boiled in sample buffer. Blotting was performed using an Immobilon-FL polyvinylidene difluoride membrane (Milipore) following the manufacturer’s directions. Rabbit anti-GlActin polyclonal antibody (31), anti-HA mouse monoclonal HA7 antibody (IgG1; Sigma-Aldrich), and mouse monoclonal anti-acetylated tubulin clone 6-11B-1 antibody (IgG2b; product T 6793; Sigma-Aldrich) were used at 1:2,500 in blocking solution (5% dry milk, 0.05% Tween 20 in Tris-buffered saline). Secondary anti-mouse isotype-specific antibodies conjugated with Alexa 488 (anti-IgG2b), Alexa 555 (anti-IgG1), and anti-rabbit Alexa 647 were used. For CWP1 staining, Alexa 647-conjugated anti-CWP1 antibody (Waterborne, New Orleans, LA) was used at 1:200. Horseradish peroxidase-linked anti-mouse or anti-rabbit antibodies (Bio-Rad) were used at 1:7,000. Multiplex immunoblots were imaged by using a Chemidoc MP system (Bio-Rad).

### Image analysis.

Segmentation analysis was performed with Imaris software (Bitplane, version 8.1). Images were processed with ImageJ (57), and figures were assembled using Adobe Illustrator.

## SUPPLEMENTAL MATERIAL

Figure S1  GL50803_8496 sequence analysis. BLAST alignment against the human proteome identified Rac1 as the top hit. Key features of GlRac are annotated, including the Q74 mutation site, predicted small GTP-binding protein domain, and CaaX site. Download Figure S1, PDF file, 0.2 MB

Figure S2  Constitutive GlRac signaling disrupts ESV organization and causes ER swelling. TEM imaging of wild-type and doxycycline-induced HA-Rac^CA^ cells 13 h p.i.e. White arrowheads, putative ESVs; yellow arrowheads, putative ER; N, nucleus. Insets are 5×-magnified views to show fine detail of ESVs. Bar = 1 µm. Download Figure S2, PDF file, 0.5 MB

Figure S3  Time course of the encystation process, revealing a role for GlRac in promoting ESV maturation. Cells expressing endogenously tagged HA-Rac or tetracycline-inducible HA-Rac^CA^ were encysted and stained for HA (green), CWP1 (ESV marker; red), and DNA (blue) every 2 h from 2 to 24 h p.i.e. Cells expressing HA-Rac^CA^ produced ESV with condensed cores earlier than wild-type Rec-expressing cells (8 h p.i.e.), indicating accelerated maturation. HA-Rac^CA^ also alters the number of ESVs formed. Bar = 5 µm. Download Figure S3, PDF file, 0.9 MB

Figure S4  Uninduced HA-Rac^CA^ cells have an intermediate phenotype due to leaky expression of HA-Rac^CA^. (A) Endogenously tagged HA-Rac, uninduced HA-Rac^CA^, and induced HA-Rac^CA^ cells were stained for HA and CWP at 8 h p.i.e. (B) Quantification of ESVs by 3D segmentation analysis showed a significant difference in the number of ESVs per cell at 8 h p.i.e. for HA-Rac^CA^-expressing cells compared with endogenously tagged HA-Rac-expressing cells. (C) ESV volumes were quantified by 3D segmentation analysis. Note that while induced HA-Rac^CA^ cells had the largest ESVs, the uninduced HA-Rac^CA^ cells had an intermediate phenotype. The *t* test showed significant differences: *, *P* < 0.05; ***, *P* < 0.001; ****, *P* < 0.0001. Bar = 5 µm. Download Figure S4, PDF file, 0.6 MB

Figure S5  Depletion of GlRac affects cell morphogenesis. In order to monitor knockdown of GlRac, a morpholino-sensitive version of HA-Rac (HA-Rac^MS^) was constructed and integrated into the *Giardia* genome. (A) The native promoter of GlRac is followed by the first 27 bp of the coding region (morpholinos are 25 bp) of GlRac, followed by the coding region of the 3× HA tag and the full GlRac coding region (see Materials and Methods in the full text). (B and C) Electroporation of *Giardia* trophozoites with an antisense GlRac morpholino suppresses translation of the genomically integrated HA-Rac^MS^ and results in 72% ± 18% depletion compared to cells transfected with control morpholino (Ctrl), as determined by Western blotting. A representative blot from five biological replicates is shown. (D) HA-Rac^MS^ localization is indistinguishable from HA-Rac. Note that depletion of GlRac in trophozoites affects cell morphology, polarity, and membrane organization and results in multinuclear cells. Bar = 5 µm. Download Figure S5, PDF file, 2 MB

Figure S6  Depletion of GlRac impairs CWP1 secretion and affects cell morphology during late encystation. (A) Control and GlRac-depleted cells were stained for HA-Rac^MS^ (green), CWP1 (red), and DNA (blue). Cells in the later part of encystation showed morphological defects, including cell protrusions, multiple and mislocalized nuclei, irregular ESVs, and sometimes fully formed cysts with a tail of trapped vesicles. Bar = 5 µm. (B) Western blot showing CWP levels in cells and in media. Note the reduced secretion of CWP in GlRac knockdown cells. Download Figure S6, PDF file, 1.9 MB

Table S1  *Giardia* has a minimal Rho-GTPase signaling system. Table S1, PDF file, 0.2 MB
