# Supplementary material for: Rac Regulates Giardia lamblia Encystation by Coordinating Cyst Wall Protein Trafficking and Secretion
Source: mBio. 2016 Aug 23;7(4):e01003-16. doi: 10.1128/mBio.01003-16 (PMC4999545; doi:10.1128/mBio.01003-16)
Supplement: Figure S1 — GL50803_8496 sequence analysis. BLAST alignment against the human proteome identified Rac1 as the top hit. Key features of GlRac are annotated, including the Q74 mutation site, predicted small GTP-binding protein domain, and CaaX site. Download [file mbo004162953sf1.pdf]

Gl50803_8496 top BLAST hit against human proteins is Rac1, Sequence ID: ref|NP_008839.2|

```
Score              Expect        Identities          Positives           Gaps
236 bits(601)      3.00E-84      109/177(62%)        135/177(76%)        0/177(0%)
GlRac  14    MIHIKAVVVGDGSVGKTCLLFVYANNSFPEDYLPTVFDNYSANVVVDNLTINIGLWDTAG  73
             M  IK VVVGDG+VGKTCLL  Y  N+FP +Y+PTVFDNYSANV+VD   +N+GLWDTAG
Rac1   1     MQAIKCVVVGDGAVGKTCLLISYTTNAFPGEYIPTVFDNYSANVMVDGKPVNLGLWDTAG  60

GlRac  74    QEDYDKLRPLSYPGAHVFLLCFSVVSSTSFANIRSKWYTEVKEYCPNVPMILVGTKYDLL  133
             QEDYD+LRPLSYP   VFL+CFS+VS   SF N+R+KWY EV+ +CPN P+ILVGTK DL
Rac1   61    QEDYDRLRPLSYPQTDVFLICFSLVSPASFENVRAKWYPEVRHHCPNTPIILVGTKLDLR  120

Rac1   134   SDEAYLAKMKEKNQSPVSDERAEEVAKEIKAIKYISCSARCQLRVKDVFDSAIRAAL  190
              D+   + K+KEK  +P++  +   +AKEI A+KY+ CSA  Q  +K VFD AIRA L
Rac1   121   DDKDTIEKLKEKKLTPITYPQGLAMAKEIGAVKYLECSALTQRGLKTVFDEAIRAVL  177
```

GlRac 218 amino Acids:

```
        10          20          30          40          50
MTSTGNEDTA  GARMIHIKAV  VVGDGSVGKT  CLLFVYANNS  FPEDYLPTVF
        60          70          80          90         100
DNYSANVVVD  NLTINIGLWD  TAGQEDYDKL  RPLSYPGAHV  FLLCFSVVSS
       110         120         130         140         150
TSFANIRSKW  YTEVKEYCPN  VPMILVGTKY  DLLSDEAYLA  KMKEKNQSPV
       160         170         180         190         200
SDERAEEVAK  EIKAIKYISC  SARCQLRVKD  VFDSAIRAAL  KNMGMMGSGT
       210
SKAGKKKDGS  GKGKCVIF
```

Q74: key residue for GTP hydrolysis is equivelent to Q61 of Rac1. Mutation to L results in Rac^{CA} form

IHI...YIS (15-169): small GTP-binding protein domain (IPR005225) according to UniProt

CaaX Prenylation Prediction:

C-terminal consensus sequence of prenylated proteins, C cysteine, a aliphatic aminoacid (G, A, V, L, I), X any aminoacid

prenylation site prediction according to GPS Lipid (http://lipid.biocuckoo.org)

**Position**	**Peptide**	**Score**	**Cutoff**	**Type**
215	DGSGKGKCVIF	10.421	6.806	S-Farnesylation: CAAX
215	DGSGKGKCVIF	5.917	3.059	S-Geranylgeranylation: CAAX

prenylation site prediction according to PrePS (http://mendel.imp.ac.at/PrePS/index.html)

**Position**	**Peptide**	**Score**	**p-value**	**Type [quality of prediction out of +++)]**
215	DGSGKGKCVIF	1.422	3.4e-04	FT - CaaX-Farnesyltransferase (++)
215	DGSGKGKCVIF	1.563	1.3e-03	GGT1 - CaaX Geranylgeranyltransferase (++)
