# Supplementary figures and images for: Rac Regulates Giardia lamblia Encystation by Coordinating Cyst Wall Protein Trafficking and Secretion
Source: mBio. 2016 Aug 23;7(4):e01003-16. doi: 10.1128/mBio.01003-16 (PMC4999545; doi:10.1128/mBio.01003-16)

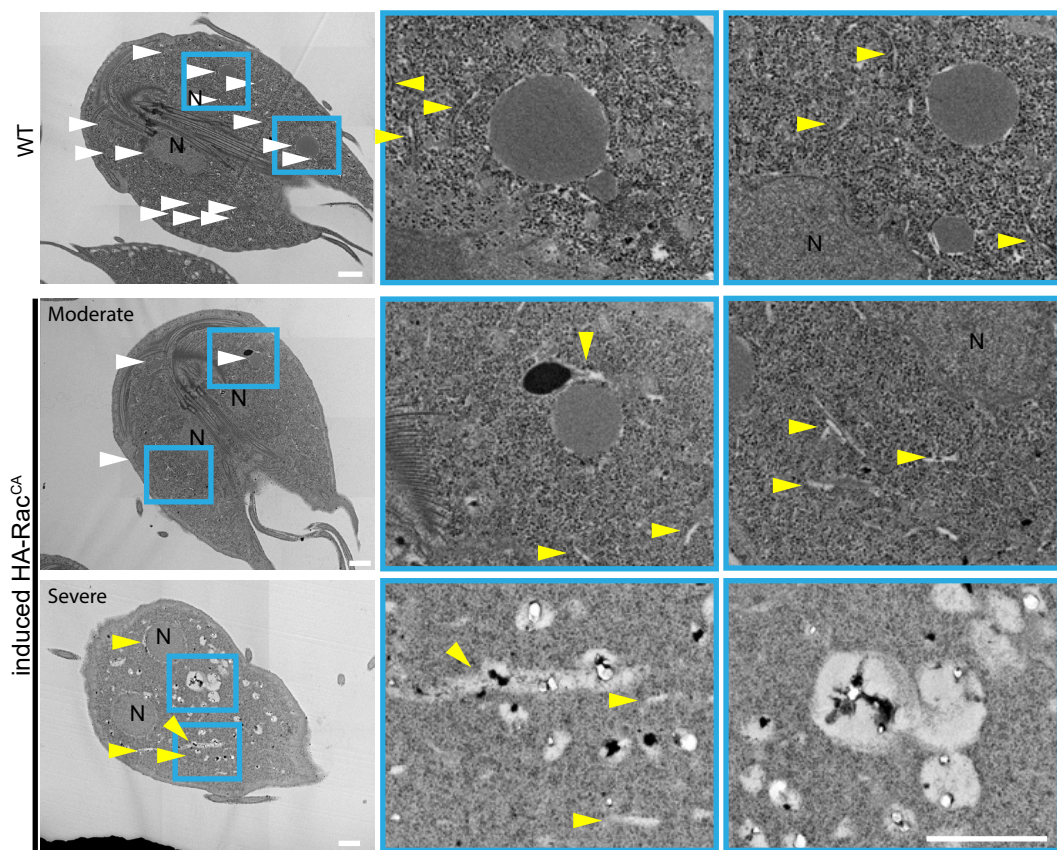

Supplement: Figure S2 — Constitutive GlRac signaling disrupts ESV organization and causes ER swelling. TEM imaging of wild-type and doxycycline-induced HA-RacCA cells 13 h p.i.e. White arrowheads, putative ESVs; yellow arrowheads, putative ER; N, nucleus. Insets are 5×-magnified views to show fine detail of ESVs. Bar = 1 µm. Download [file mbo004162953sf2.pdf]

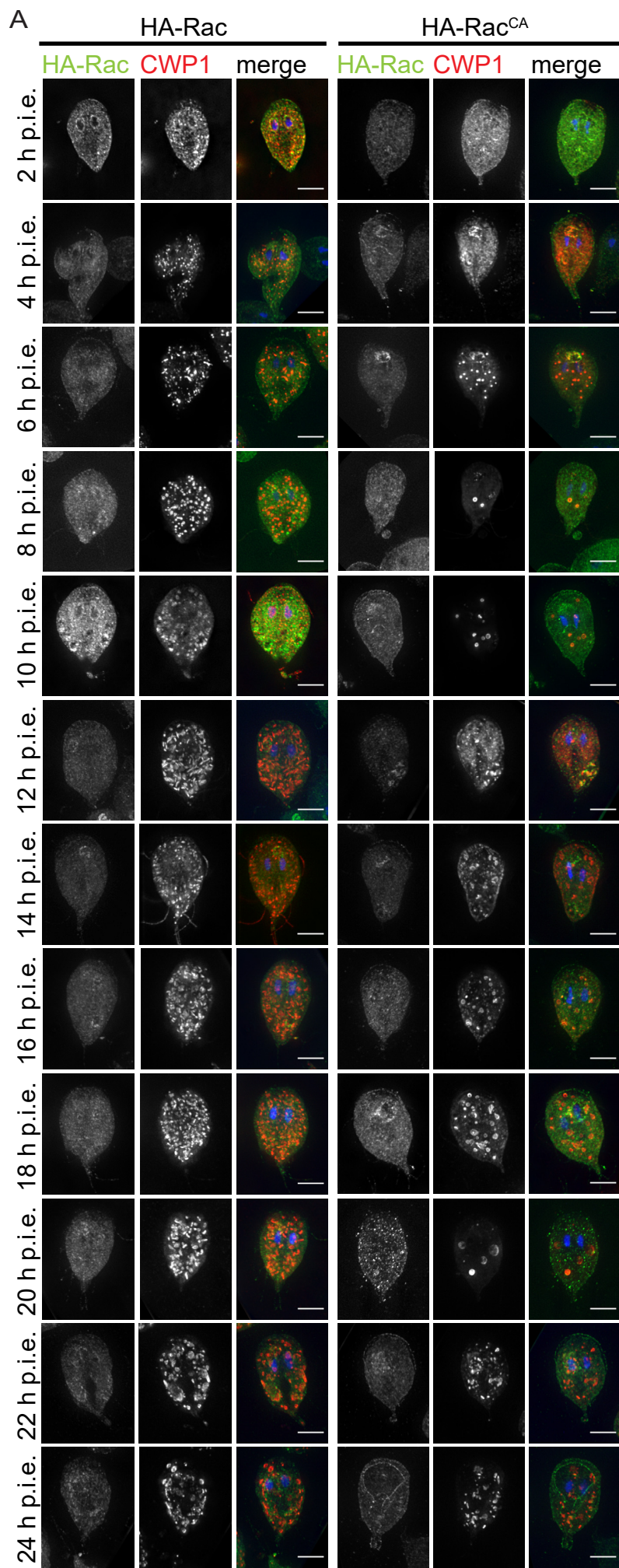

Supplement: Figure S3 — Time course of the encystation process, revealing a role for GlRac in promoting ESV maturation. Cells expressing endogenously tagged HA-Rac or tetracycline-inducible HA-RacCA were encysted and stained for HA (green), CWP1 (ESV marker; red), and DNA (blue) every 2 h from 2 to 24 h p.i.e. Cells expressing HA-RacCA produced ESV with condensed cores earlier than wild-type Rec-expressing cells (8 h p.i.e.), indicating accelerated maturation. HA-RacCA also alters the number of ESVs formed. Bar = 5 µm. Download [file mbo004162953sf3.pdf]

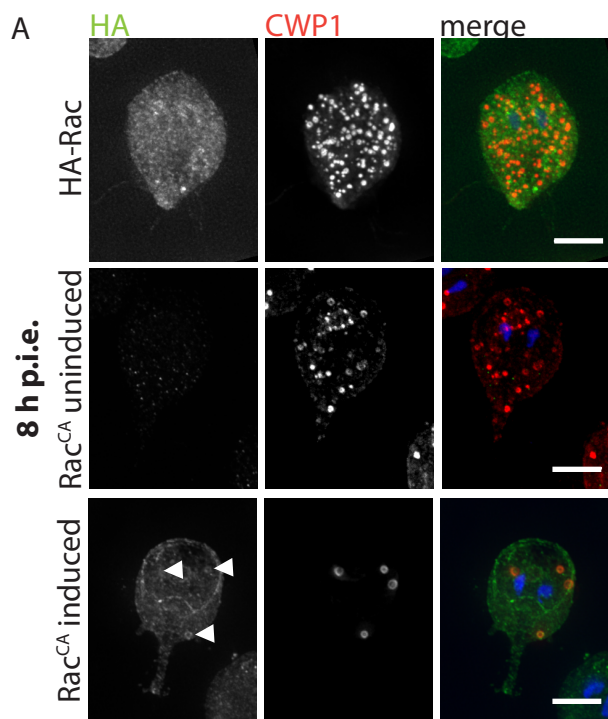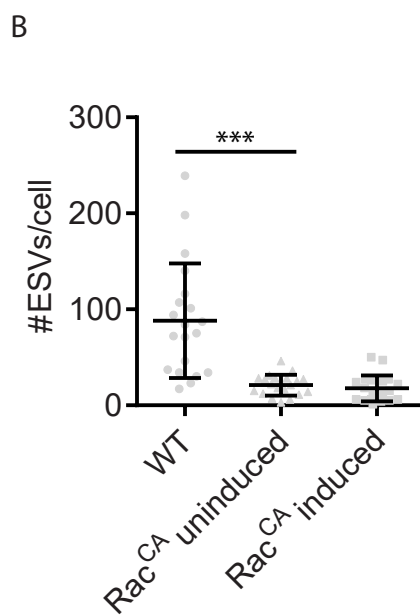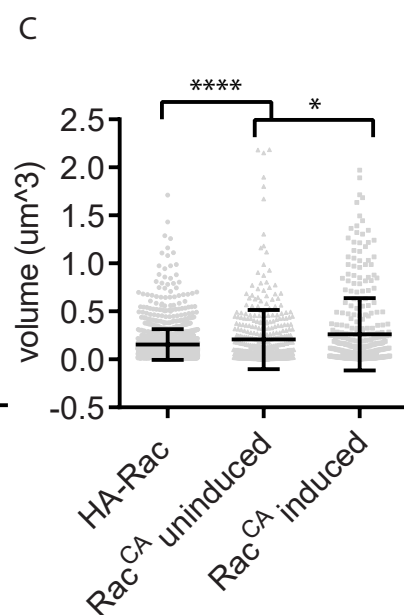

Supplement: Figure S4 — Uninduced HA-RacCA cells have an intermediate phenotype due to leaky expression of HA-RacCA. (A) Endogenously tagged HA-Rac, uninduced HA-RacCA, and induced HA-RacCA cells were stained for HA and CWP at 8 h p.i.e. (B) Quantification of ESVs by 3D segmentation analysis showed a significant difference in the number of ESVs per cell at 8 h p.i.e. for HA-RacCA-expressing cells compared with endogenously tagged HA-Rac-expressing cells. (C) ESV volumes were quantified by 3D segmentation analysis. Note that while induced HA-RacCA cells had the largest ESVs, the uninduced HA-RacCA cells had an intermediate phenotype. The t test showed significant differences: *, P < 0.05; ***, P < 0.001; ****, P < 0.0001. Bar = 5 µm. Download [file mbo004162953sf4.pdf]

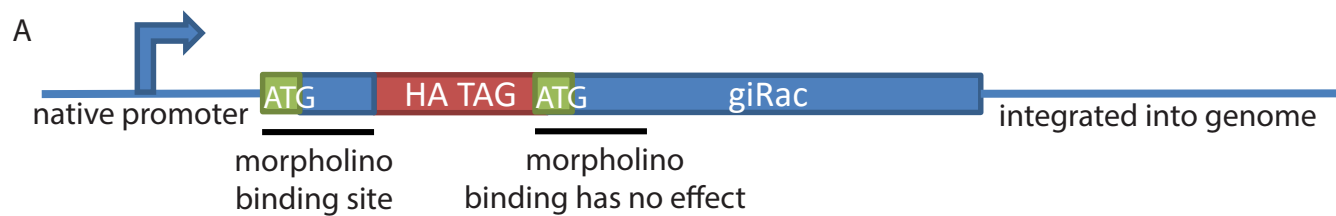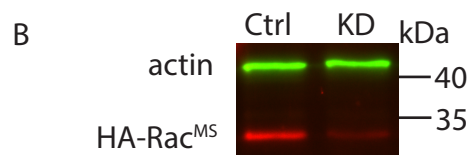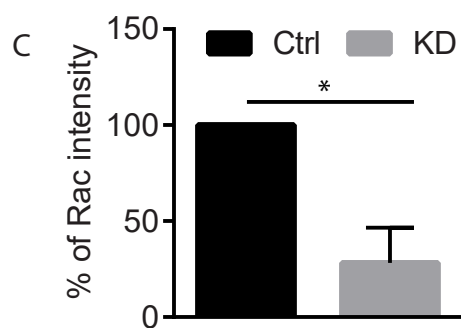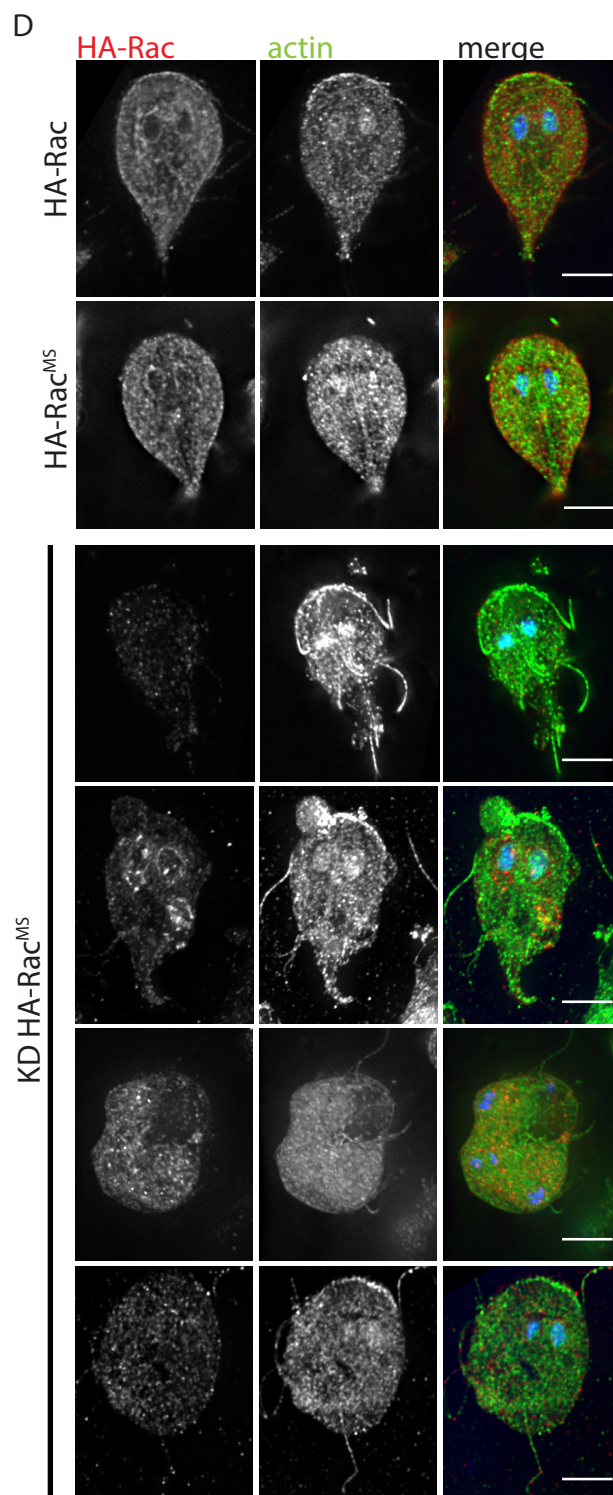

Supplement: Figure S5 — Depletion of GlRac affects cell morphogenesis. In order to monitor knockdown of GlRac, a morpholino-sensitive version of HA-Rac (HA-RacMS) was constructed and integrated into the Giardia genome. (A) The native promoter of GlRac is followed by the first 27 bp of the coding region (morpholinos are 25 bp) of GlRac, followed by the coding region of the 3× HA tag and the full GlRac coding region (see Materials and Methods in the full text). (B and C) Electroporation of Giardia trophozoites with an antisense GlRac morpholino suppresses translation of the genomically integrated HA-RacMS and results in 72% ± 18% depletion compared to cells transfected with control morpholino (Ctrl), as determined by Western blotting. A representative blot from five biological replicates is shown. (D) HA-RacMS localization is indistinguishable from HA-Rac. Note that depletion of GlRac in trophozoites affects cell morphology, polarity, and membrane organization and results in multinuclear cells. Bar = 5 µm. Download [file mbo004162953sf5.pdf]

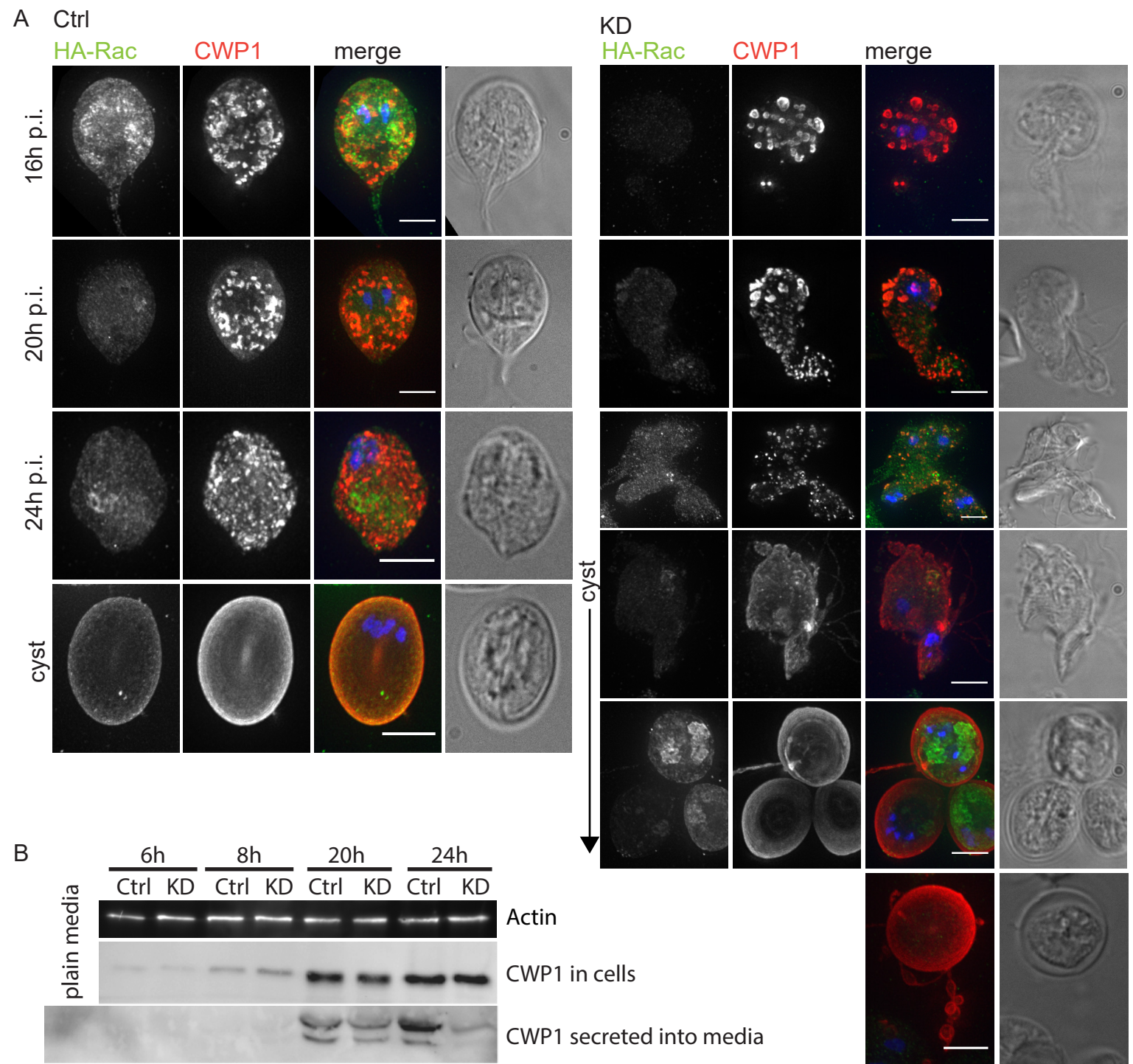

Supplement: Figure S6 — Depletion of GlRac impairs CWP1 secretion and affects cell morphology during late encystation. (A) Control and GlRac-depleted cells were stained for HA-RacMS (green), CWP1 (red), and DNA (blue). Cells in the later part of encystation showed morphological defects, including cell protrusions, multiple and mislocalized nuclei, irregular ESVs, and sometimes fully formed cysts with a tail of trapped vesicles. Bar = 5 µm. (B) Western blot showing CWP levels in cells and in media. Note the reduced secretion of CWP in GlRac knockdown cells. Download [file mbo004162953sf6.pdf]
