# Supplementary material for: Rac Regulates Giardia lamblia Encystation by Coordinating Cyst Wall Protein Trafficking and Secretion
Source: mBio. 2016 Aug 23;7(4):e01003-16. doi: 10.1128/mBio.01003-16 (PMC4999545; doi:10.1128/mBio.01003-16)
Supplement: Table S1 — Giardia has a minimal Rho-GTPase signaling system. [file mbo004162953st1.pdf]

Table 1. Giardia has a minimal Rho GTPase signaling system

Annotation	GeneID	MW	# in Humans
**Rho Family GTPase:**			23 (1)
Rac/Rho Like GTPase	GL50803_8496	23903	
**Activators (GEFs):**			60+ (1)
ELMO/CED12 domain	GL50803_95878	77027	
ELMO/CED12 domain	GL50803_17246	23986	
DOCK domain	GL50803_114201	411622	
DOCK domain	GL50803_114209	347902	
**Negative Regulators:**			70+ (1)
Rac GAP	GL50803_14608	69334	
Rac GAP	GL50803_13550	21724	
Rac GAP	GL50803_10056	22659	
GDI	GL50803_2925	22,110	
**Effectors:**			
PAK Kinase	GL50803_2796	64237	7 (1)
Actin Binding Proteins	Not found		10+ (1)

1. **Bustelo XR, Sauzeau V, Berenjeno IM.** 2007. Gtp-Binding Proteins of the Rho/Rac Family: Regulation, Effectors and Functions in Vivo. Bioessays **29:**356-370.
